# Revealing the Role of Beesioside O from *Actaea vaginata* for the Treatment of Breast Cancer Using Network Pharmacology, Molecular Docking, and Molecular Dynamics Simulation

**DOI:** 10.3390/ijms26052283

**Published:** 2025-03-04

**Authors:** Shuyang Li, Juan Lu, Hongwei Xue, Yang Lou, Jia Liu, Yutian Wang, Haifeng Wu, Xi Chen

**Affiliations:** 1 State Key Laboratory for Quality Ensurance and Sustainable Use of Dao-di Herbs, Institute of Medicinal Plant Development, Chinese Academy of Medical Sciences, Peking Union Medical College, Beijing 100193, China; 15563999708@163.com (S.L.); jlu@implad.ac.cn (J.L.); 15297318065@163.com (H.X.); louyang0727@163.com (Y.L.); wytqingya@163.com (Y.W.); 2 Department of Pharmaceutical Analysis, Heilongjiang University of Chinese Medicine, Harbin 150040, China; lj200929@126.com

**Keywords:** breast cancer, BO, anticancer, network pharmacology, molecular docking, molecular dynamics simulation

## Abstract

Breast cancer remains a leading cause of malignancy-related mortality among women, with rising global incidence. While surgical intervention is effective for early-stage breast cancer, drug therapy is indispensable, particularly for triple-negative breast cancer, where treatment options are still limited. *Actaea vaginata*, a traditional Chinese medicinal herb, has been historically applied for inflammatory conditions, including pharyngitis and stomatitis. However, its antitumor potential remains under-reported. In this study, a cycloartane triterpene saponin, beesioside O (BO), was isolated from this plant. Its antitumor activity was evaluated in vitro. Its potential therapeutic mechanisms were elucidated through network pharmacology. BO exhibited substantial potency in inhibiting breast cancer cells. Network pharmacology analysis uncovered 179 potential pharmacological targets of BO, which were predominantly concentrated in pathways, such as pathways in cancer, the PI3K-Akt signaling pathway, and chemical carcinogenesis receptor activation. Molecular docking analysis indicated that STAT3 exhibited minimal binding energy with BO. Additionally, molecular dynamics simulations verified the conformational stability of the BO-STAT3 complex. Western blot analysis demonstrated that STAT3 was downregulated following administration. These results imply that BO may exhibit a multi-target, synergistic therapeutic effect against breast cancer, with STAT3 recognized as a pivotal target. This study demonstrates the potential of BO for development as a chemotherapeutic agent for breast cancer treatment. It lays the groundwork for further exploration of BO’s bioactivity and provides valuable insights into its molecular mechanisms in breast cancer therapy.

## 1. Introduction

According to the research conducted by the International Agency for Research on Cancer (IARC), breast cancer was the most prevalent cancer worldwide in 2020, with over 2.26 million new cases reported globally [1]. In China, breast cancer has also emerged as the most prevalent type of cancer, with statistics indicating that it is the leading cause of cancer among women and that it significantly reduces survival rates [2]. Metastasis, rather than the primary tumor, is the leading cause of mortality in individuals with breast cancer [3]. Surgery remains a crucial method for managing breast cancer, especially in its initial phases [4]. However, in low- and middle-income countries, breast cancer in women is often diagnosed at later stages due to limited medical resources and advancements. By this time, the tumor has already spread, making it less responsive to surgical interventions [5,6]. For patients in the advanced stages, where cancer has spread to remote organs, pharmacological treatment becomes a vital and unavoidable approach [7]. The significance of anti-breast-cancer medications lies in their use in both surgical and non-surgical treatment contexts. Surgery can lead to physical changes, affecting the patient’s overall well-being. For early-stage breast cancer, existing surgical methods can be paired with medication to reduce the tumor size as much as possible, minimizing damage to the patient [8,9]. Although several therapeutic agents, including tamoxifen, anastrozole, trastuzumab, and capecitabine, are presently utilized in the clinical management of breast cancer, many of these drugs exhibit limitations due to their receptor specificity. Given the absence of receptor expression, strong metastatic capability, and resistance to conventional endocrine treatments and targeted therapies, triple-negative breast cancer (TNBC) poses substantial challenges in therapeutic decision making, resulting in highly restricted treatment options [10]. Consequently, the pursuit of innovative targeted drugs, immunotherapeutic approaches, and more accurate diagnostic techniques to enhance treatment efficacy for breast cancer, particularly TNBC, has become a key area of contemporary research. In this regard, revisiting natural compounds and traditional medicines may offer fresh perspectives and potential breakthroughs to address the constraints of current treatment strategies.

*Actaea vaginata* (Maxim.) J. Compton is the sole species of the genus *Actaea* in the tribe Cimicifugeae and is distributed in the southwest and northwest of China. It exhibits anti-inflammatory properties and is effective against conjunctivitis, stomatitis, pharyngitis, and enteritis, serving as an alternative to *Coptis chinensis* Franch in Shaanxi province [11]. Previous chemical investigations of this plant have disclosed the presence of triterpenoids, alkaloids, and phenolic acids. Among these constituents, cycloartane triterpene glycosides exhibited diverse biological activities, including anti-complement, antitumor, and cholinesterase enzyme inhibitory effects [12]. However, as a constituent of the cycloartane triterpene glycosides in *A. vaginata*, an investigation of the anticancer properties of BO has yet to be conducted.

Signal transducer and activator of transcription 3 (STAT3) is a cytoplasmic inactive transcription factor that falls into the category of the STAT protein family. It comprises six functional regions, including an oligomerization domain at the N-terminus, an alpha-helical domain for intermolecular interactions, a core DNA-binding region, a connecting domain that regulates the stability of DNA binding, a canonical Src homology 2 (SH2) motif, and a C-terminal activation domain. STAT3 conveys signals from the cell membrane to the nucleus and actively engages in the transcriptional regulation of numerous genes’ expression. Research has found that STAT3 serves as the crossroad of various oncogenic signaling pathways, and its dysregulation plays an oncogenic role in both solid and hematological malignancies [13,14,15,16]. An increasing number of STAT3 inhibitors are under investigation for anticancer purposes.

Herein, the entire *A. vaginata* plant was extracted and purified to obtain BO using semi-preparative liquid chromatography. In vitro activity screening of BO against various tumor cell lines revealed its potent anticancer effects on the human breast cancer cell lines MCF-7 and MDA-MB-231. Using network pharmacology, potential targets of this compound were predicted for breast cancer therapy, and key targets were identified. Molecular docking was employed to forecast interactions with these key targets, and the results were further confirmed through molecular dynamics simulations and Western blot analysis. This study evaluates the antitumor activity of BO for the first time and confirms that BO extracted from *A. vaginata* exhibits therapeutic potential against various types of breast cancer, including TNBC, by modulating STAT3, a non-surface receptor target. As a natural product, BO possesses a relatively complex structure, which still holds value for further structural modification and simplification. Therefore, this discovery has the potential to introduce a novel class of therapeutic agents into clinical breast cancer treatment, expand drug options for TNBC management, and reduce the risk of drug resistance associated with prolonged monotherapy.

## 2. Results

### 2.1. Chemistry

**Figure 1:**
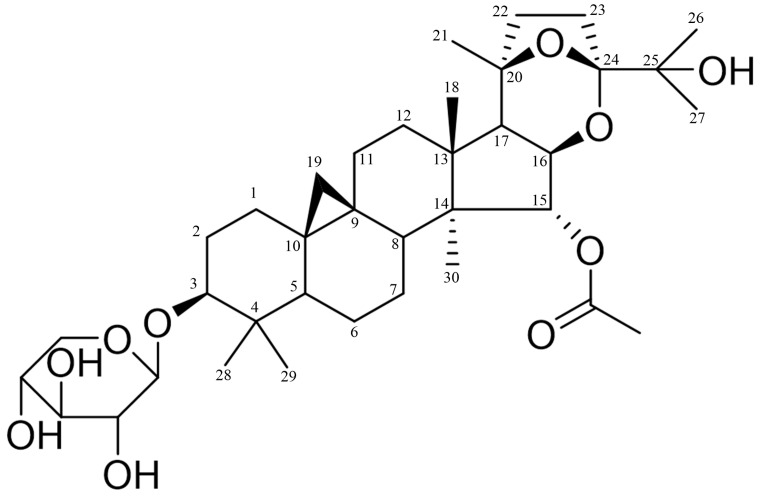
Chemical structure of BO. Cartesian coordinates of BO’s atoms; the 3D structure can be seen in Files S1 and S2.

BO was isolated as a white amorphous powder, was easily soluble in methanol and pyridine, and gave a positive Liebermann–Burchard reaction, which suggested that the compound could probably be a triterpene derivative. HRESI-MS (positive) *m*/*z*: 685.3932 [M + Na]^+^. Its molecular weight was determined to be 662, and its molecular formula was speculated to be C_37_H_58_O_10_. ^1^H NMR (600 MHz, Pyridine-*d*_5_) δ_H_: 1.22 (1H, m, H-1a), 1.58 (1H, m, H-1b); 1.94 (1H, m, H-2a), 2.35 (1H, m, H-2b), 3.51 (1H, dd, *J* = 11.4, 4.2 Hz, H-3), 1.31 (1H, m, H-5), 0.67 (1H, q, *J* = 12.5 Hz, H-6a), 1.52 (1H, m, H-6b), 1.02 (1H, m, H-7a), 1.33 (1H, m, H-7b), 1.76 (1H, dd, *J* = 12.6, 4.2 Hz, H-8), 1.07 (1H, m, H-11a), 2.09 (1H, m, H-11b), 1.66 (1H, m, H-12a), 1.05 (1H, m, H-12b), 5.57 (1H, br, s, H-15), 4.46 (1H, brd, *J* = 7.2 Hz, H-16), 1.82 (1H, d, *J* = 6.6 Hz, H-17), 1.58 (3H, s, H-18), 0.27 (1H, d, *J* = 3.6 Hz, H-19a), 0.49 (1H, d, *J* = 3.6 Hz, H-19b), 1.33 (3H, s, H-21), 2.00 (1H, m, H-22a), 1.65 (1H, m, H-22b), 2.59 (1H, m, H-23a), 1.98 (1H, m, H-23b), 1.64 (3H, s, H-26), 1.47 (3H, s, H-27), 1.39 (3H, s, H-28), 1.08 (3H, s, H-29), 1.25 (3H, s, H-30), 4.84 (1H, d, *J* = 7.2 Hz, H-1′), 4.02 (1H, t, *J* = 8.4 Hz, H-2′), 4.15 (1H, t, *J* = 8.4 Hz, H-3′), 4.22 (1H, m, H-4′), 3.74 (1H, t, *J* = 10.8 Hz, H-5′a), 4.35 (1H, dd, *J* = 10.8, 4.8 Hz, H-5′b), 2.08 (3H, s, 15-COCH_3_). ^13^C-APT NMR (150 MHz, Pyridine-d_5_) δ_C_: 32.8 (C-1), 30.5 (C-2), 88.9 (C-3), 41.7 (C-4), 47.9 (C-5), 21.4 (C-6), 26.4 (C-7), 48.2 (C-8), 20.1 (C-9), 26.8 (C-10), 26.5 (C-11), 33.6 (C-12), 47.0 (C-13), 50.3 (C-14), 86.6 (C-15), 80.3 (C-16), 51.6 (C-17), 22.0 (C-18), 31.0 (C-19), 82.8 (C-20), 26.2 (C-21), 40.5 (C-22), 29.0 (C-23), 111.1 (C-24), 72.4 (C-25), 25.9 (C-26), 25.6 (C-27), 25.0 (C-28), 15.8 (C-29), 14.1 (C-30), 108.0 (C-1′), 76.0 (C-2′), 79.0 (C-3′), 71.6 (C-4′), 67.6 (C-5′), 170.6 (15-COCH_3_), 21.4 (15-COCH_3_). The above compound data are consistent with the literature [17]. The study cited here employed high-resolution FABMS to obtain an *m*/*z* value of 663.4089 (calc. 663.4108) for [M + H]^+^, which led to the determination of the molecular formula of BO as C_37_H_58_O_10_. Furthermore, IR spectrum analysis revealed that BO was a glycoside compound with an ester group. The planar structure of BO was elucidated through comprehensive NMR spectroscopic analysis, which included ^1^H NMR, ^13^C NMR, ^1^H-^1^H COSY, HMQC, and HMBC spectra. The structural formula is shown in Figure 1.

### 2.2. ADMET Prediction Results for BO

**Figure 2:**
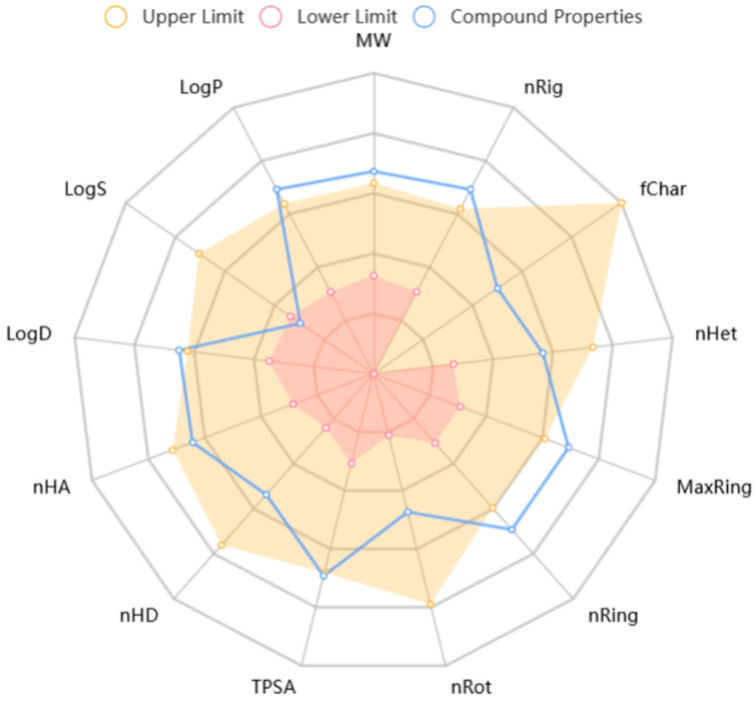
The ADMET properties of BO.

In silico measurements were conducted for physicochemical descriptors. MW (molecular weight), nRig (number of rings), fChar (formal charge), nNet (number of heteroatoms), MaxRing (number of atoms in the biggest ring), nRing (number of rigid bonds), nRot (number of rotatable bonds), TRSA (topological polar surface area), nHD (number of hydrogen bond donors), nHA (number of hydrogen bond acceptors), LogD (the LogP at pH = 7.4), LogS (the logarithm of the aqueous solubility value), and LogP (the logarithm of the n-octanol/water distribution coefficient) were the indices used to evaluate the pharmacokinetic properties. As Figure 2 shows, the red and yellow radar charts represent the predicted minimum and maximum limits, respectively. If the compound properties are not between the upper and lower limits, this indicates that there are deficiencies in the physical and chemical properties of BO. BO’s LogP, LogD, TPSA, nRing, MaxRing, nRig, and MW values all exceed the upper limits of the ideal model, while its LogS value is below the lower limit of the ideal model; the remaining parameters fall within the range of the ideal model. The connection between glucose and the complex multi-ring structure is the cause of the unsatisfactory prediction results. These properties cause a relatively high MW (662.4, optimal: 100–600), which has a negative impact on BO’s in vivo absorption and water solubility. The high molecular weight is also the reason for BO’s overly high LogP (3.486, optimal: 0–3) and LogD values (3.2, optimal: 1–3) and overly low LogS value (−4.497, optimal: −4–0.5 log mol/L). Additionally, this structural feature excessively increases BO’s TPSA (144.14, optimal: 0–140), potentially having a negative effect on BO’s intracellular transport. Moreover, a suitable nHet (10, optimal range: 1–15) provides BO with sufficient nHA (10, optimal range: 0–12) and nHD (4, optimal range: 0–7). This enables BO to potentially form hydrogen bonds with amino acid residues of target proteins [18]. The abundance of hydrogen bond acceptors and donors grants BO the potential for strong binding affinity with its target. Reducing the molecular weight of BO through modification will have better pharmacological effects.

Based on the predicted physicochemical properties of BO, its potential effects on in vivo bioavailability and pharmacokinetics can be inferred as follows. Absorption: Due to its higher molecular weight, lower LogS, and larger TPSA, the oral bioavailability of this compound is likely to be low. Distribution: The high LogP value indicates that the compound is well distributed in lipid environments; however, its distribution in tissues may be restricted due to its higher polar surface area and molecular weight. Metabolism: The complex structure of the compound (such as its multiple rings and stereocenters) may lead to intricate metabolic pathways, potentially involving various enzymes for its metabolism. Excretion: The higher molecular weight and polarity suggest that the compound is likely excreted primarily through the kidneys, although the exact excretion pathways require further experimental validation.

BO has the problems of an excessive number of inner rings and high molecular rigidity, which indicate the future optimization path of BO. For example, the sugar moiety at C-3 can be removed, and modifications can be made to the aglycone by introducing functional groups that enhance protein affinity. Additionally, the hydroxyl group at C-25 can also be modified.

### 2.3. Screening of the In Vitro Antitumor Activity of BO

SW480, Huh7, 4T1, SHSY5Y, HepG2, MCF-7, and MDA-MB-231 cells were seeded into 96-well plates at a density of 10^4^ cells per well. Once the cells reached 70% confluence, they were treated with the compound at different concentrations for 24 h. The efficacy of the treatment was assessed using the MTT assay. Each experiment was performed in triplicate, and related results are summarized in Table 1. For Huh7 and SW480, due to the IC_50_ values in vitro exceeding 100 µM, BO did not show any therapeutic value. The IC_50_ value for the SHSY5Y cell line was determined to be 86.91 µM, suggesting that BO exhibits limited therapeutic potential and may not be a promising candidate for further investigation. During the screening of HepG2, the IC_50_ value of 21.17 µM indicated that BO had a certain therapeutic value for this type of liver cancer. During the screening of the breast cancer cell lines MCF-7 and MDA-MB-231, BO exhibited significant therapeutic potential, with IC_50_ values below 20 µM for both cell lines. Therefore, we conclude that breast cancer cells exhibit the greatest sensitivity to BO. Based on the favorable IC_50_ values, breast cancer was selected as the focus for further investigation.

### 2.4. Verification of BO’s Anti-Breast-Cancer Function in Cellular Models

**Figure 3:**
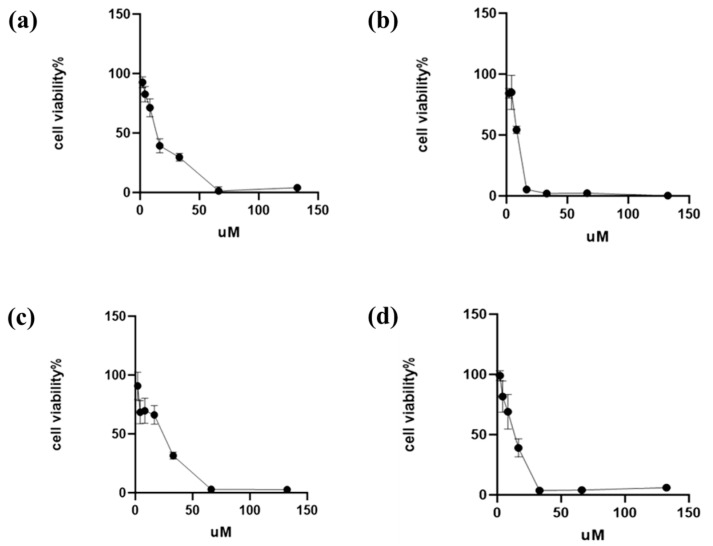
Effect of BO on the viability of breast cancer cells. BO at concentrations from 1.5 µM to 130 µM was employed to test its effective dosage range and IC_50_. (**a**) BO for MCF-7 in 24 h, IC_50_ = 13.83 µM; (**b**) BO for MCF-7 in 48 h, IC_50_ = 8.19 µM; (**c**) BO for MDA-MB-231 in 24 h, IC_50_ = 16.52 µM; and (**d**) BO for MDA-MB-231 in 48 h, IC_50_ = 11.91 µM. BO was capable of reducing breast cancer cell viability in a dose- and time-dependent fashion.

**Figure 4:**
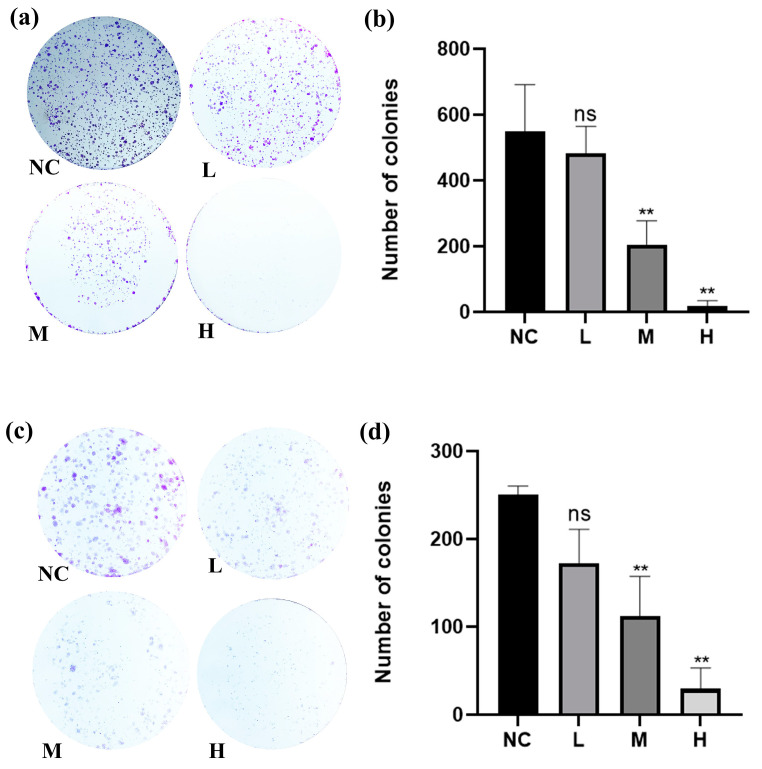
The results of the colony formation assay with 7 µM, 14 µM, and 28 µM in MCF-7 and 8 µM, 16 µM, and 32 µM in MDA-MB-231, administered for 24 h and cultured for 14 days. (**a**) The figures for MCF-7 at different concentrations and (**b**) statistical results of BO’s colony formation assay in MCF-7 in the form of a bar chart. As the concentration was increased, the proliferative capacity of MCF-7 cells was reduced, and their tumorigenicity was significantly inhibited. (**c**) The figures of MDA-MB-231 at different concentrations and (**d**) statistical results of BO’s colony formation assay in MDA-MB-231 in the form of a bar chart; the proliferative capacity of MDA-MB-231 cells was reduced, and their tumorigenicity was significantly inhibited. All values are presented as the mean ± SD, with *n* = 3. “ns” *p* ≥ 0.05, ** *p* < 0.01 compared with the control group.

**Figure 5:**
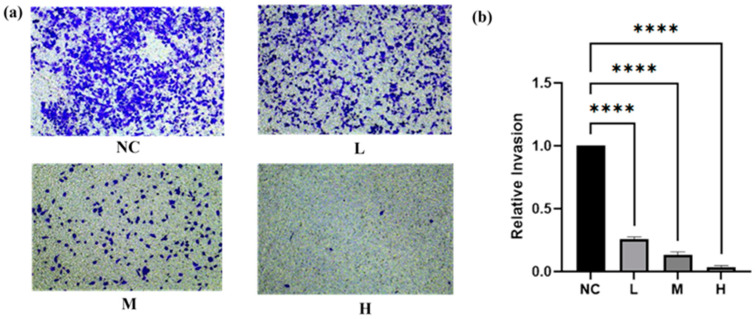
The representative results of the transwell invasion experiment with 8 µM, 16 µM, and 32 µM for MDA-MB-231, administered and cultured for 24 h. (**a**) The figures of MDA-MB-231 at different concentrations and (**b**) statistical results of BO’s invasion experiment with MDA-MB-231 in the form of a bar chart. The experimental results demonstrated that as the dosage increased, the number of MDA-MB-231 cells that migrated to the lower chamber significantly decreased. This indicates that BO inhibits the migration of MDA-MB-231 cells, and this inhibitory effect is positively correlated with the drug dosage. All data are presented as the mean ± SD, with *n* = 3. **** *p* < 0.0001 compared with the control group.

To verify the effects of BO against breast cancer, a range of cellular biological assays were conducted using the MCF-7 and MDA-MB-231 cell lines. As illustrated in Figure 3, BO was capable of reducing breast cancer cell viability in a dose- and time-dependent fashion, with estimated IC_50_ values of approximately 13.83 µM and 16.52 µM in the MCF-7 and MDA-MB-231 cell lines, respectively, within 24 h. When the administration time was extended to 48 h, the IC_50_ values of the MCF-7 and MDA-MB-231 cell lines came to 8.19 µM and 11.91 µM, respectively. In addition, the IC_50_ value in MCF-7 was smaller than that in MDA-MB-231, indicating that MCF-7 was more sensitive to BO than MDA-MB-231. A colony formation assay showed that BO can inhibit the growth of MDA-MB-231 and MCF-7 (Figure 4), and this inhibition was enhanced by increasing the drug concentration. MDA-MB-231, as a TNBC cell line, exhibits a high metastatic potential. Compared with the normal control (NC) group, BO was also found to suppress the invasiveness of MDA-MB-231 cell lines in transwell assays. This inhibition was also intensified as the drug concentration increased (Figure 5).

### 2.5. Discovery of Targets and Pathways Associated with Breast Cancer

**Figure 6:**
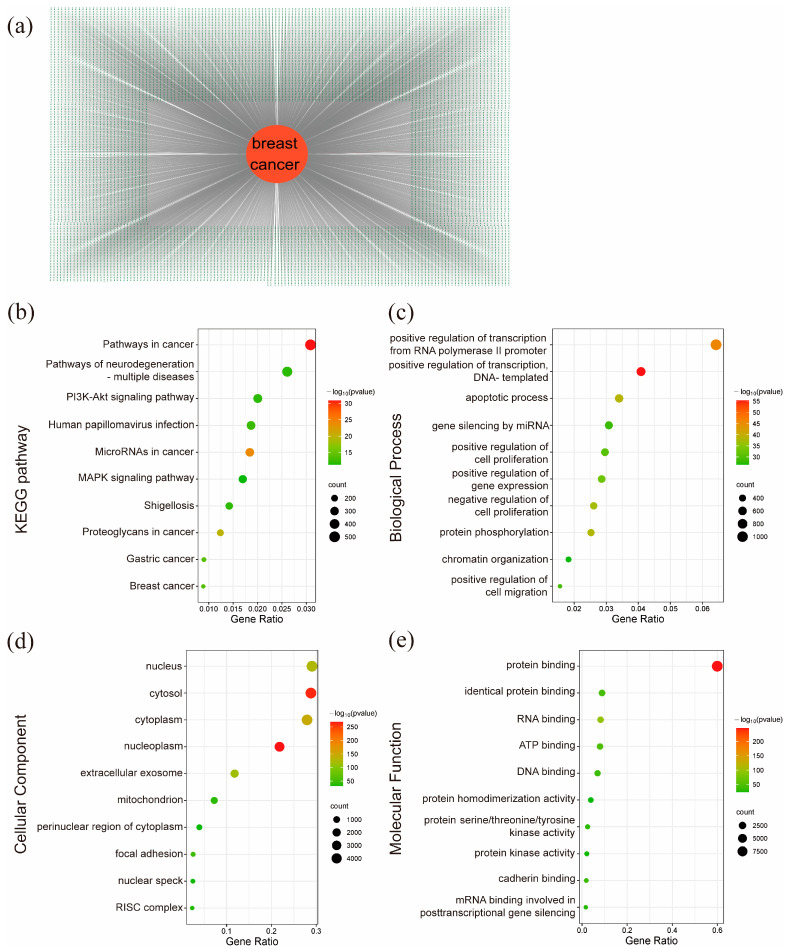
Related targets, GO functions, and KEGG pathways of breast cancer. (**a**) A total of 17,044 targets of breast cancer and (**b**) bubble diagram of the top 10 pathways in breast cancer in the KEGG pathway enrichment analysis. These are pathways in cancer, pathways of neurodegeneration—multiple diseases, the PI3K-Akt signaling pathway, human papillomavirus infection, microRNAs in cancer, the MAPK signaling pathway, Shigellosis, proteoglycans in cancer, gastric cancer, and breast cancer. Given that pathways in cancer AND pathways of neurodegeneration—multiple diseases function as overarching integrative networks, the ‘PI3K-Akt Signaling Pathway’ emerges as a critical and specific pathway playing a pivotal role in breast cancer progression. (**c**) Bubble diagram of the top 10 BP enrichments in breast cancer; (**d**) bubble diagram of the top 10 CC enrichments in breast cancer; and (**e**) bubble diagram of the top 10 MF enrichments in breast cancer.

To identify probable mechanisms and therapeutic targets for breast cancer, 17,044 breast-cancer-related targets were collected from the TTD, GeneCards, and OMIM databases. GO and KEGG pathway enrichment analyses were conducted to unveil potential therapeutic pathways, and Figure 6b–e shows 10 significantly enriched items. The results showed that ‘positive regulation of transcription from RNA polymerase II promoter’, ‘protein binding’, and ‘nucleus’ were the most significantly enriched items among biological processes (Table S3), molecular functions (Table S4), and cellular components (Table S5), respectively. As depicted in Figure 6b, ‘Pathway in Cancer’ contains the maximum number of targets (551 counts, Table S6), followed by ‘Pathways of Neurodegeneration—Multiple Diseases’ and the ‘PI3K-Akt Signaling Pathway’. According to the KEGG website, ‘Pathways in Cancer’ is a comprehensive collection of cancer-related pathways, encompassing several classical signaling routes, including the ‘PI3K-Akt pathway’, and it serves as an integrative framework rather than a singular pathway. Similarly, ‘Pathways of Neurodegeneration—Multiple Diseases’ represents a broad category related to multiple neurological disorders. Given that both of these pathways function as overarching integrative networks, the ‘PI3K-Akt Signaling Pathway’ emerges as a critical and specific pathway playing a pivotal role in breast cancer progression.

### 2.6. Recognition of BO-Specific Targets and In-Depth Pathway Examination for Breast Cancer Treatment

**Figure 7:**
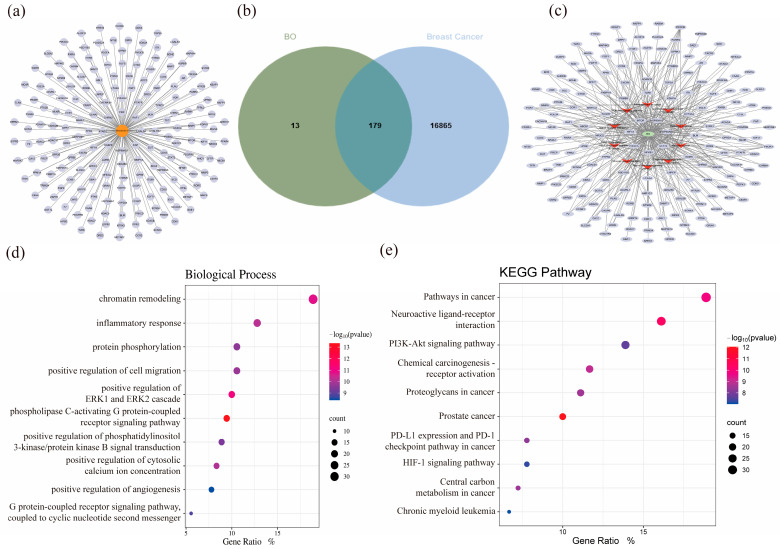
Identification of BO-related targets. (**a**) A total of 192 targets of BO from different databases; related targets are visualized as gray ellipses. (**b**) Overlapping targets of BO and breast cancer, which means that BO may achieve its treatment of breast cancer through these targets. (**c**) BO–target–pathway network, where mint green circular nodes represent BO, scarlet V-shaped nodes represent pathways, and gray-blue ellipses represent the enriched targets. This includes the following pathways: pathways in cancer, neuroactive ligand–receptor interaction, PI3K-Akt signaling pathway, chemical carcinogenesis–receptor activation, proteoglycans in cancer, prostate cancer, PD-L1 expression and PD-1 checkpoint pathways in cancer, HIF-1 signaling pathway, central carbon metabolism in cancer, and chronic myeloid leukemia. Compared with the findings in Section 2.5, the PI3K-Akt signaling pathway is the most possible pathway for BO’s treatment of breast cancer. (**d**) Bubble diagram of the top 10 BP enrichments in overlapping targets. (**e**) Bubble diagram of the top 10 KEGG pathway enrichments in overlapping targets; these pathways are the same as the results in Figure 7c.

To identify the specific targets through which BO interacts with breast cancer cells, we conducted a comprehensive analysis; as shown in Figure 7a and Table S7, a total of 192 BO targets were identified using ChemMapper, Super-PRED, and Swiss Target Prediction. Of these, 179 overlapping targets between BO and breast cancer were selected for further analysis (Figure 7b, Table S8). To elucidate the possible biological functions and KEGG pathways of BO in breast cancer cells, the 179 overlapping targets were analyzed using the Wei Sheng Xin cloud platform. The 10 most prominently enriched BP and KEGG pathway terms are visualized in Figure 7d,e (Tables S9 and S10). These enrichment analyses showcased the prospective therapeutic routes through which BO affects breast cancer cells. Among these, ‘Pathways in Cancer’ and ‘Neuroactive ligand–receptor interaction’ were the most prominent; however, ‘Neuroactive ligand–receptor interaction’ plays a major role in the nervous system, and the ‘Pathways in Cancer’ was described in Section 2.5. Other pathways, such as ‘Prostate cancer’ and ‘Chronic myeloid leukemia’, are not applicable when describing breast cancer. Therefore, we hypothesize that the ‘PI3K-Akt Signaling Pathway’, ‘Chemical carcinogenesis-receptor activation’, ‘Proteoglycans in cancer’, ‘HIF-1 signaling pathway’, ‘PD-L1 expression and PD-1 checkpoint pathway in cancer’, and ‘Central carbon metabolism in cancer’ may play critical roles in BO’s anticancer activity against breast cancer. A recent study showed that the PI3K-Akt pathway and HIF-1 signaling pathway play vital roles in the survival and development of breast cancer. The PI3K-Akt pathway transmits signals from GPCRs, regulating downstream targets, such as mTOR, and, thereby, controlling breast cancer cell growth, apoptosis, and drug resistance [19,20,21]. The HIF-1 signaling pathway has great significance in drug resistance, cancer stem-like properties, and the hypoxia characteristics of breast cancer. It can achieve the metabolic reprogramming of breast cancer cells and change their carbon metabolism, which makes breast cancer worse [22,23,24,25,26,27,28,29,30]. Additionally, according to the pathway map in KEGG, the HIF-1 signaling pathway and PI3K-Akt pathway are closely related. Specifically, this can be demonstrated by the regulation of STAT3 and key targets in the PI3K-AKT-MTOR pathway, such as PIK3CA and MTOR, which, in turn, modulate the crucial target HIF1A in the HIF-1 signaling pathway [31,32,33,34,35]. In addition, the HIF-1 signaling pathway shows the regulation of STAT3 after IL-6 stimulation [36], which shows the potential of BO in regulating immunity to achieve tumor treatment [37,38]. Other significant pathways, including the PD-L1 expression and PD-1 immune checkpoint pathways [39,40,41,42], have also been extensively studied in breast cancer. The central carbon metabolism in cancer includes various pathways, such as the TCA cycle, nucleotide synthesis, amino acid synthesis, and the Warburg effect. The central carbon metabolism is also closely linked to both the PI3K-Akt and HIF-1 signaling pathways, significantly influencing the occurrence and progression of breast cancer [43,44,45,46,47].

BP enrichment analysis of the targets revealed that BO’s therapeutic biological processes in breast cancer are primarily concentrated in the G-protein coupled receptor (GPCR) signaling pathways, inflammatory response, chromatin remodeling, and protein phosphorylation. GPCRs are a large family of membrane proteins that serve as the most abundant cell surface receptors and are involved in various downstream signaling pathways, including those related to immune inflammation and protein phosphorylation. They also cause changes in chromatin and other substances in the nucleus [48,49,50]. Compared with the results of the biological process analysis for breast cancer in Section 2.5, ‘chromatin’, ‘protein phosphorylation’, and ‘positive regulation of cell migration’ will be the possible key processes for BO in breast cancer treatment. These processes are among the top ten biological processes in the analysis in Section 2.5, which indicated that the biological processes through which BO exerts its therapeutic effects on breast cancer also play a significant role in the pathogenesis of the disease itself. This implies that BO may interact with fundamental processes of the pathogenesis of breast cancer.

To more clearly illustrate the relationship between the targets and pathways involved in BO’s treatment of breast cancer, the BO–target–pathway network is visualized in Figure 7c using Cytoscape 3.10.3. The network includes 190 nodes (179 targets and 10 pathways after excluding targets that were not within the network system) and 379 edges. These signaling pathways and targets are likely key mechanisms through which BO treats breast cancer, including pathways, such as those of STAT3, NFKB1, PI3K-Akt, PD-L1 expression, and the PD-1 checkpoint. Currently published articles provide evidence that these target pathways are valuable for the treatment of breast cancer.

**Figure 8:**
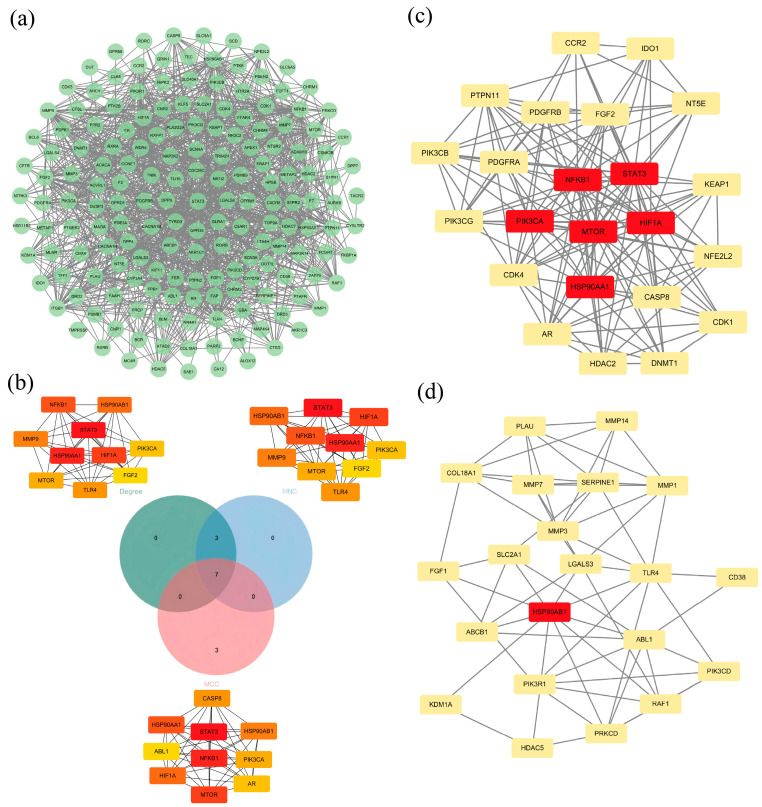
Analysis of BO-related targets. (**a**) PPI network of overlapping targets (172 nodes and 1059 edges after excluding targets that were not within the network system); green ellipses represent different targets. (**b**) Hub targets: The intersection of the top 10 targets in terms of their Degree, MCC, and MNC in the PPI network. Ranked according to their Degree scores from highest to lowest, the top 10 are STAT3, HSP90AA1, HIF1A, NFKB1, HSP90AB1, MMP9, TLR4, MTOR, PIK3CA, and FGF2; the top 10 in terms of MNC are STAT3, HSP90AA1, HIF1A, NFKB1, HSP90AB1, MMP9, TLR4, MTOR, PIK3CA, and FGF2; the top 10 in terms of MCC are STAT3, NFKB1, MTOR, HSP90AA1, HIF1A, HSP90AB1, CASP8, PIK3CA, AR, and ABL1. To eliminate the limitations of using a single computational method, we took the intersection of the aforementioned results, which yielded seven targets: HIF1A, STAT3, HSP90AA1, HSP90AB1, PIK3CA, MTOR, and NFKB1. (**c**) Cluster 1 (score 13.636) of the MCODE analysis included 23 nodes, 150 edges, and STAT3, MTOR, HSP90AA1, HIF1A, NFKB1, and PIK3CA. (**d**) Cluster 2 (score 6.200) of the MCODE analysis included 21 nodes, 62 edges, and HSP90AB1.

To study the essential targets of BO in breast cancer more thoroughly, the PPI network was assessed and represented using the STRING database and the Cytoscape 3.10.3 software (Figure 8a). The STRING settings were configured as follows: organism, *Homo sapiens*; minimum required interaction score, and medium confidence (0.400). Using CytoHubba, the top 10 nodes were selected based on three estimation methods: Degree, MCC, and MNC. The combination of multiple estimation methods effectively eliminates the potential flaws and unreasonable results that may arise from relying on a single estimation method. Venn diagram analysis was then used to identify seven core targets, namely, HIF1A, STAT3, HSP90AA1, HSP90AB1, PIK3CA, MTOR, and NFKB1 (Figure 8b). Two prominent modules were revealed by the MCODE analysis, with scores of 13.636 (cluster 1) and 6.200 (cluster 2), respectively. These modules include seven core targets, as shown in Figure 8c,d. By comparing the results from different algorithms and taking the intersection, we found that this intersection perfectly matches the seven targets, with no targets being excluded. Therefore, we can confidently conclude that these seven targets truly hold a central position.

### 2.7. Subcellular Localization Expression Analysis of Key Genes

According to HPA, experimental data on the subcellular localization of hub targets (HSP90AA1, HSP90AB1, STAT3, PIK3CA, HIF1A, MTOR, and NFKB1) were gathered. The sub-localization of HSP90AA1 and HSP90AB1 in human cells certified that HSP90AA1 and HSP90AB1 proteins resided in the cytosol based on the evidence provided by 62 types of breast cancer cell lines, including MCF-7 and MDA-MB-231. In addition, the other core genes are scattered in various zones of cells, such as vesicles, the Golgi apparatus, nucleoplasm, and nuclear bodies. In addition, hub genes are distributed in 62 breast cancer cell lines, which cover different subtypes of breast cancer (Table 2). In Section 3.5, the GO analysis demonstrated that the key cellular components involved in the pathogenesis of breast cancer include the ‘nucleus’, ‘cytosol’, and ‘nucleoplasm’. This result aligns with the subcellular localization analysis, indicating that most of the core targets are concentrated in these three areas. This suggests that these three components, especially the nucleoplasm and cytosol, may play key roles in the therapeutic effects of BO in breast cancer.

### 2.8. Molecular Docking of the Compound and Targets

Molecular docking is a core computational method employed in drug development, and it is designed to examine interactions between molecules, such as ligands and receptors, in order to forecast their binding patterns and affinities. The binding interactions between potential active compounds and critical targets were studied using AutoDockTools-1.5.6, Discovery Studio 2016, and the PyMOL software. The protein architectures of hub targets were acquired online from RCSB PDB, and they included PIK3CA (PDB-ID: 5DXT), STAT3 (PDB-ID: 6NUQ), HSP90AA1 (PDB-ID: 2YK9), HSP90AB1 (PDB-ID: 3NMQ), MTOR (PDB-ID: 4JSX), HIF1A (PDB-ID: 4H6J), and NFKB1 (PDB-ID: 1SVC). For proteins with inhibitors, the original inhibitor was removed, and molecular docking of BO was conducted at the inhibitor binding site. For crystal structures without inhibitors, blind docking was performed after analyzing the protein structure. The binding scores were recorded after completing docking. The binding scores are shown in Table 3.

**Figure 9:**
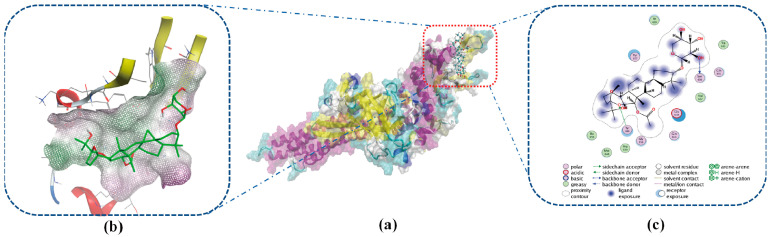
Molecular docking results. (**a**) Docking of BO- STAT3 (PDB ID: 6 nuq) with −6.78 kcal/mol and a Ki value of 10.71 µM. (**b**) Docking site of BO and STAT3. (**c**) Two-dimensional interactions of BO and STAT3; the result reveals 13 amino acid residues participating in the formation of the docking environment. They are six polar residues (Ser636, Gln635, Tyr640, Gln644, Gly656, and Tyr657), one acidic residue (Glu638), and six hydrophobic residues (Trp623, Val637, Pro639, Met648, and Ile653). The hydrophobic pocket regions formed by Met 648, Tyr 640, Ile 653, Pro639, and Glu 638 are important for STAT3. BO can promote the inhibitory effect by forming H-bonds and filling space in these areas.

Notably, a lower value indicated a stronger binding ability. The docking results showed that not all of the values were less than −5 kcal/mol. The lowest result appeared for STAT3, with −6.78 kcal/mol, and the Ki value was 10.71 µM. This result was derived based on the substitution of the original inhibitor ligand, ensuring a high level of reliability. This means that STAT3 may be the key target of BO when treating breast cancer. As illustrated in Figure 9c, BO was tightly connected to STAT3 through hydrogen bonds that formed with Ser 636 and Tyr 640. The BO ligand demonstrates a high degree of exposure during docking, indicating a favorable binding propensity toward STAT3. The macromolecular structure of STAT3 reveals 13 amino acid residues participating in the formation of the docking environment. Among these, six polar residues (Ser636, Gln635, Tyr640, Gln644, Gly656, and Tyr657) and one acidic residue (Glu638) with exceptionally high exposure levels were identified. The remaining six residues are hydrophobic (Trp623, Val637, Pro639, Met648, Ile653, and Ile659).

This spatial distribution suggests that polar interactions, in addition to hydrogen bonding, play a significant role in mediating the binding affinity between BO and STAT3. In the design of STAT3 inhibitors by Gao et al. [51], it was reported that the formation of hydrogen bonds between Glu638 and Tyr640 enhances binding activity. The utilization of hydrogen bonding or hydrophobic interactions to fill the hydrophobic pocket region formed by Met 648, Tyr 640, Ile 653, and Pro639 can improve activity. BO primarily exhibits low-polarity structural exposure in this hydrophobic pocket and forms hydrogen bonds. This feature enhances BO’s affinity for STAT3.

### 2.9. Molecular Dynamics Simulation

**Figure 10:**
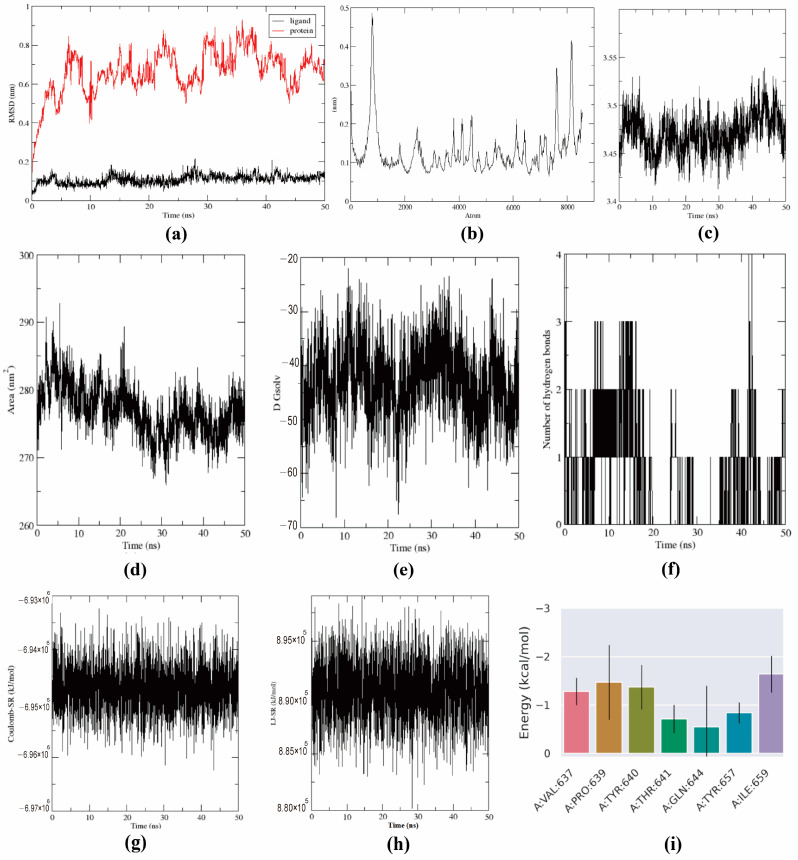
Molecular dynamics simulations of BO-STAT3. (**a**) RMSD fluctuations of the protein and ligands. RMSD curves remain in fairly constant stages over 50 ns of MD simulations, demonstrating the credibility of the docking conformations. (**b**) RMSF of the protein, which shows a lower value, suggesting that the atoms of the protein are stable. (**c**) Rg fluctuation of the protein. During the 50 ns of the simulation, the Rg shows little variation over time, indicating that the BO-STAT3 complex maintains a relatively compact and stable structure. (**d**) SASA analysis of the protein. (**e**) Solvation free energy is near −40 kcal/mol at 50 ns, suggesting the stability of the complex. (**f**) Number of hydrogen bonds between the protein and BO. The most H-bonds that can be formed in the BO-STAT3 complex is 4. (**g**) Coulomb-SR energy between the protein and BO is near −6.95 × 10^6^ at 50 ns, suggesting the stability of the complex. (**h**) LJ-SR energy is near 8.9 × 10^5^ at 50 ns, suggesting the stability of the complex. (**i**) Energy contributions of each residue—VAL637, PRO639, TYR640, and ILE659—may play a crucial role in free-energy binding.

Molecular dynamics simulations were carried out to support the docking results by examining the molecular motions of BO when bound to the ligand. The root-mean-square deviation (RMSD) is an indicator used to measure the stability of molecular structures in molecular dynamics simulations. When the RMSD fluctuations of both ligands and proteins are small, it usually means that the simulation system maintains a relatively stable state during the simulation process without significant relative displacement or conformational changes. As evidenced by Figure 10a, the RMSD curves remained in fairly constant stages over 50 ns of MD simulations, demonstrating the credibility of the docking conformations. Figure 10b shows a lower value, suggesting that the atoms of the protein are stable. The radius of gyration (Rg) can characterize the compactness of a protein’s structure during the simulation process. As shown in Figure 10c, the Rg of BO and STAT3 remains relatively small throughout the simulation. Moreover, during the 50 ns of the simulation, the Rg shows little variation over time, indicating that the BO-STAT3 complex maintains a relatively compact and stable structure. Hydrogen bonds are some of the strongest non-covalent interactions, and the greater the number of hydrogen bonds, the stronger the binding effect. As shown in Figure 10f, BO was observed to form four hydrogen bonds with STAT3. The SASA parameter of the protein and the solvation free energy value oscillated near −40 kcal/mol and around 270 nm^2^, as shown in Figure 10d,e, demonstrating that the complex is relatively stable. The Coulomb-SR energy and LJ-SR energy between the protein and STAT3 are shown in Figure 10g,h. Figure 10i depicts amino acid residues whose energy contribution values fall below −1 kcal/mol, implying that VAL637, PRO639, TYR640, and ILE659 play a crucial role in free-energy binding. In the calculation of the binding free energy, the GBSA parameters mainly include VDWAALS (describing non-bonded interactions between molecules, primarily involving intermolecular attractive forces), EEL (reflecting electrostatic interactions between molecules, including attraction or repulsion between positive and negative charges), EGB (considering interactions between the solvent and polar groups on the protein surface), ESURF (considering interactions between the solvent and non-polar regions), DELTA G gas (representing the energy of the molecule in the gas phase, including VDWAALS and EEL), and solvation DELTA G solv (combining EGB and ESURF and reflecting the overall interaction between the solvent and both the protein and small molecule). DELTA TOTAL (total binding free energy) integrates all of these factors and is used to assess the stability of the binding between the protein and the small molecule. As shown in Table 4, the GBSA results from the simulation were favorable, indicating that the binding can occur spontaneously. Focusing on the ∆TOTAL values in Table 5, the results of PBSA were similar to those of GBSA, which showed the strong possibility of spontaneous binding between STAT3 and BO.

### 2.10. Differential Gene Analysis

**Figure 11:**
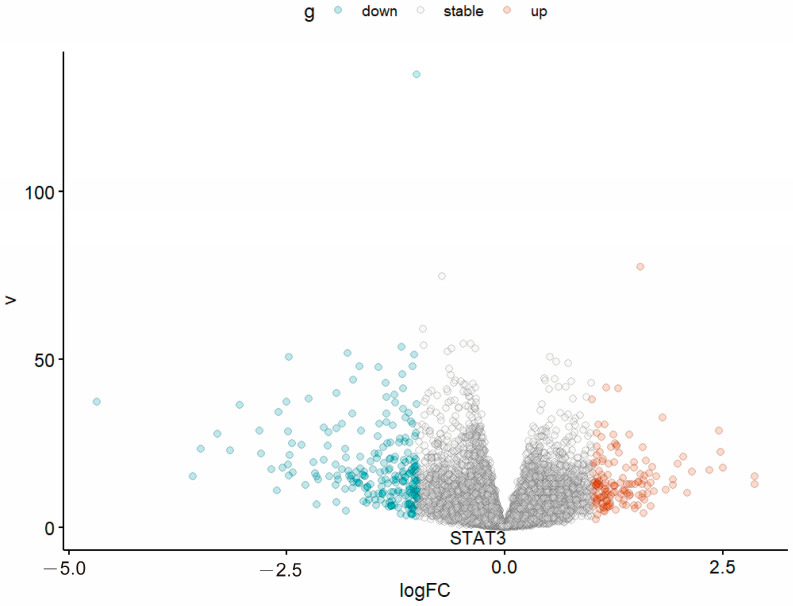
The volcano plot of STAT3 in TNBC and non-TNBC. The “v” on the longitudinal axis represents −log10 (*p* value). The figure shows no significant differences in STAT3 in TNBC and non-TNBC patients, so BO can treat these two different subtypes of breast cancer cell lines.

Special attention was given to STAT3 to evaluate its potential value in breast cancer. To explore the differences in STAT3 between different types of breast cancer, we chose to analyze TNBC and non-TNBC samples in the GEO database (GSE76275) using R packages. The volcano plot (Figure 11) demonstrated no substantial alterations in STAT3 expression between TNBC and non-TNBC (logFC > 1 or logFC < −1, *p* < 0.05). This can explain why BO can affect both MCF-7 and MDA-MB-231.

### 2.11. Western Blot Verification

**Figure 12:**
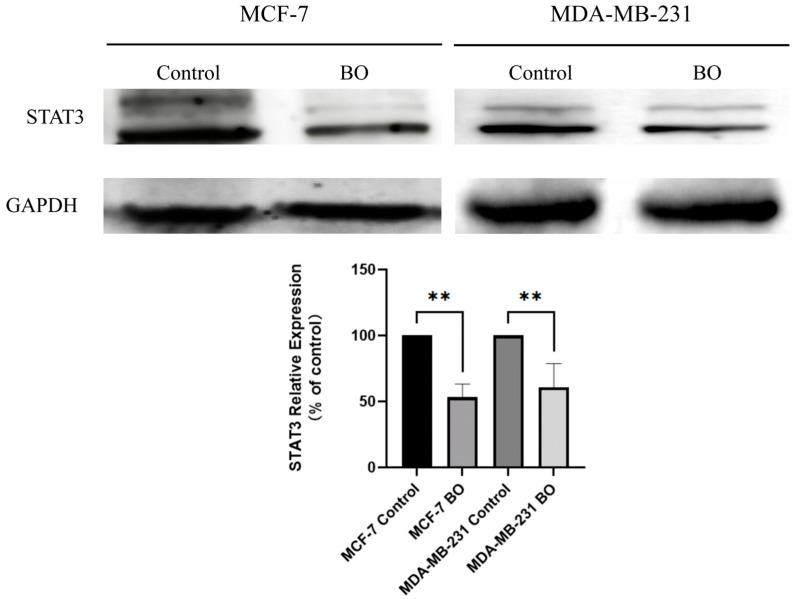
Western blot analysis of MCF-7 and MDA-MB-231 after treatment with BO (20 µM, 24 h). The expression of STAT3 decreased after administration. All data are presented with *n* = 3, ** *p* < 0.01.

STAT3 protein expression was analyzed with a Western blot (Figure 12). Compared with the control group, the relative expression of STAT3 in MCF-7 decreased to 53%, while STAT3 decreased to 60% in MDA-MB-231 after treatment with 20 µM BO for 24 h. After administration, BO significantly downregulated STAT3 in the MDA-MB-231 and MCF-7 breast cancer cell lines.

## 3. Materials and Methods

### 3.1. Reagents

All chemicals of reagent grade were obtained from commercial sources. DMEM medium (12100–500 mL, Solarbio, Beijing, China), Cell Counting Kit-8 (CCK-8, CK04-5000T, Solarbio, Beijing, China), PBS (P1020, Solarbio, Beijing, China), 4% PFA (P1110, Solarbio, Beijing, China), RIPA lysis buffer (R0010, Solarbio, Beijing, China), crystal violet (C0121–100 mL, Solarbio, Beijing, China), matrigel (M8371–1 mL, Solarbio, Beijing, China), and protease inhibitor mixture (P6730-1mL, Solarbio, Beijing, China) were brought from Solarbio^®^ Life Sciences (Beijing, China). Fetal bovine serum (FBS, ST30-3302, Adenbach, Germany) was obtained from PAN-Biotech GmbH (Adenbach, Germany). In addition, 10% sodium dodecyl sulfate-polyacrylamide gel electrophoresis (DLW201-3, Tsingke, Beijing, China) was obtained from Beijing Tsingke Biotech Co., Ltd. (Beijing, China). The cECL Western Blot Kit (CW0048M, Cowin, Suzhou, China), Tris-Glycine SDS Buffer (pH8.3, 10×) (CW0045S, Cowin, Suzhou, China), TBST (pH8.0, 10×) (CW0043S, Cowin, Suzhou, China), and Tris-Glycine Transfer Buffer (pH8.3, 10×) (CW0044S, Cowin, Suzhou, China) were purchased from Jiangsu Cowin Biotech Co., Ltd. (Suzhou, China). QuickBlock^™^ Western primary antibody diluent (P0256–500 mL, Beyotime, Shanghai, China) and pancreatic enzyme cell digestion solution (0.25% trypsin, containing phenol red, without EDTA) (C0207, Beyotime, Shanghai, China) were bought from Beyotime Biotechnology (Shanghai, China).

### 3.2. Cell Lines

The human breast cancer cell lines MCF-7 and MDA-MB-231, 4T1 murine mammary carcinomas, the human liver cancer cell lines Huh7 and HepG2, the human colorectal adenocarcinoma cell line SW480, and the human neuroblastoma cell line SH-SY5Y were purchased from the Cell Resource Center, Institute of Basic Medical Sciences, Chinese Academy of Medical Sciences.

### 3.3. Plant Material

The plant *A. vaginata* was collected in August 2020 from the Qinling Mountains, Shaanxi Province, China, and was identified by Prof. Yulin Lin at the Institute of Medicinal Plant Development. A voucher specimen has been stored in the laboratory of the Institute of Medicinal Plant Development (No. AV-2020-08-10).

### 3.4. Separation and Identification of Compounds

**Figure 13:**
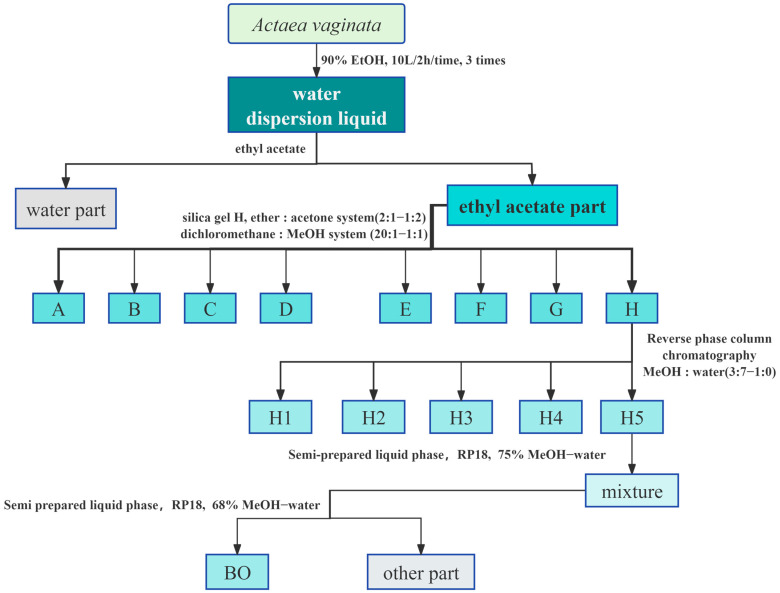
The extraction and purification of BO.

After drying and crushing 3.8 kg of the whole *A. vaginata* herb, it was extracted three times with 90% ethanol, 10 L each time, for 2 h per extraction. Filtration was performed on the extract, followed by vacuum-assisted concentration of the filtrate, yielding a crude extract. The crude extract was dispersed in water and extracted with ethyl acetate. It was then separated using a silica gel H column with gradient elution using petroleum ether–acetone (2:1~1:2) and dichloromethane–methanol systems (20:1~1:1). The fractions with the same Rf values were combined through thin-layer chromatography, yielding eight fractions labeled A-H. The H fraction was further separated using a reverse-phase C-18 column with a methanol–water gradient (3:7~1:0), yielding 5 subfractions (H1−H5). Subfraction H5 was purified using semi-preparative liquid chromatography with 75% methanol–water under the conditions of 203 nm and 2 mL/min, resulting in a mixture. This mixture was purified a second time with 68% methanol–water, resulting in the isolation of BO (*t*_R_ = 43.00 min). The relevant flowchart is presented in Figure 13. The structure of BO was identified using a chemical method, HRESI-MS, and NMR.

### 3.5. In Silico ADMET Study of BO

To profile the BO based on its physicochemical properties, determine the underlying mechanisms of its anticancer effects, and provide insightful directions for future development, we employed the ADMETLab 2.0 online platform (https://admetmesh.scbdd.com/, accessed on 3 January 2025). In the ADMETLab 2.0 online platform, we selected the “ADMET Evaluation” option under the “Services” menu and inputted the SMILES string of the BO compound to perform the calculation.

### 3.6. In Vitro Efficacy Screening Assay

To explore whether BO has antitumor activity, we selected the SW480, Huh7, 4T1, SHSY5Y, HepG2, MCF-7, and MDA-MB-231 tumor cell lines for BO activity screening. The specific operations were as follows:

#### 3.6.1. Cell Culture Conditions

All cells were cultured at 37 °C and 5% CO_2_ with DMEM medium supplemented with 10% (*v*/*v*) FBS and 1% (*v*/*v*) penicillin–streptomycin. In this case, the medium was refreshed every 2–3 days, and cells were subcultured once they attained 80% confluence.

#### 3.6.2. Cell Cytotoxicity Assay

All cells were seeded into 96-well plates at an approximate density of about 1 × 10^4^ per well and then maintained in a stable-temperature incubator at 37 °C until cultures reached 70% confluence. The culture medium was removed and re-added with a cell culture medium containing different concentrations of BO from 1.5 µM to 130 µM to proceed with incubation for 24 h or 48 h. The control group was maintained in a culture medium alone. Following incubation, the medium was removed, and 100 μL of a 10% CCK-8 dilution solution was added. After 30 min of incubation, the absorbance of each well was recorded at 450 nm using a microplate reader. Cell viability was calculated according to the following formula:

Cell viability (%) = (A_s_ − A_b_) × 100%/(A_c_ − A_b_)

where A_s_ is the experimental well, A_c_ is the control well, and A_b_ is the blank well.

#### 3.6.3. Colony Formation Assay

First, 1000 cells per well were seeded into 6-well plates and shaken to ensure they were evenly distributed. After cell attachment, the drug was added at concentrations of 7 µM, 14 µM, and 28 µM to MCF-7 and 8 µM, 16 µM, and 32 µM to MDA-MB-231 for treatment, and culturing was continued for 15 days, with regular replacement of the culture medium. The wells were washed with PBS, fixed with 4% PFA, stained with crystal violet, and then cleaned to remove the crystal violet. Photos were taken of the 6-well plates to record the results.

#### 3.6.4. Transwell Invasion Assays

Cancer cells were seeded in transwells featuring 8 µm pore membranes (353097, FALCON) plated with 20 μg/mL matrigel. The lower chambers were loaded with a medium enriched with 10% FBS. The invasion of MDA cells was assessed following a 24 h incubation.

In all invasion assays, cells that traversed to the underside of the membranes were immobilized using 4% PFA, stained with crystal violet, captured via light microscopy, and enumerated across multiple high-power fields (HPFs). The concentration of BO was the same as that in Section 2.6.

### 3.7. Gathering of BO and Breast-Cancer-Associated Target Genes

BO-related targets and breast-cancer-related targets were retrieved from various public databases, such as SwissTargetPrediction (https://www.expasy.org/resources/swisstargetprediction, accessed on 16 October 2024), Super-PRED (https://prediction.charite.de/, accessed on 16 October 2024), Therapeutic Target Database (TTD, https://db.idrblab.net/ttd/, accessed on 16 October 2024), ChemMapper (http://www.lilab-ecust.cn/chemmapper/index.html, accessed on 16 October 2024), GeneCards (https://www.genecards.org/, accessed on 16 October 2024), and Online Mendelian Inheritance in Man (OMIM, https://omim.org/, accessed on 16 October 2024). All target gene symbols were normalized using the UniProt database.

### 3.8. Protein–Protein Interaction (PPI) Network Construction and Central Target Analysis of the BO–Breast Cancer Interaction

To identify the common targets of BO and breast cancer, the intersecting targets were filtered and visualized using the Wei Sheng Xin cloud platform (http://www.bioinformatics.com.cn/, accessed on 20 October 2024). The PPI network was built using the STRING (12.0) database with Homo sapiens as the organism and a medium confidence score of 0.4. The network was then visualized and analyzed with Cytoscape (3.9.0) while utilizing the cytoHubba and MCODE plugins. The hub targets were subsequently displayed using a Venn Diagram.

### 3.9. GO and KEGG Pathway Enrichment Analyses

Gene Ontology (GO) enrichment analysis and Kyoto Encyclopedia of Genes and Genomes (KEGG) enrichment analysis of breast-cancer-related targets and BO’s targets in breast cancer were conducted using the enrichment dot bubble function on the Wei Sheng Xin cloud platform (accessed on 2 November 2024).

### 3.10. Pathway Map Analysis of Breast Cancer and BO-Associated Targets

The intricate interactions of BO within breast-cancer-related pathways were depicted using the KEGG Mapper tool on the KEGG website (https://www.kegg.jp/, accessed on 2 November 2024).

### 3.11. Assessment of Clinical Significance

We downloaded RNAseq data and clinical data on breast cancer from The Cancer Genome Atlas (TCGA, https://portal.gdc.cancer.gov/, accessed on 4 November 2024) database and Gene Expression Omnibus (GEO, https://www.ncbi.nlm.nih.gov/geo/, accessed on 4 November 2024) database. The analysis was finished using Gene Expression Profiling Interactive Analysis (GEPIA, http://gepia.cancer-pku.cn/index.html, accessed on 5 November 2024) and limma packages in R.

### 3.12. Molecular Docking

AutoDockTools 1.5.6, Discovery Studio 2016, and the PyMOL 2.4.0 software were utilized to examine the interaction between active compounds and target proteins. The 2D structures of the compounds were generated using ChemDraw 20.0 and converted into 3D structures with minimal energy using the Chem3D 20.0 software. The 3D protein structures were obtained from the Protein Data Bank (PDB, http://www.rcsb.org/, accessed on 4 January 2025). AutoDockTools 1.5.6 was employed to remove the original ligands from proteins, perform hydrogenation of the receptor proteins, and save them in PDBQT format. The compounds were similarly saved in PDBQT format after adding hydrogens. The Kollman and Gasteiger methods were used to process atomic charges. The docking position was where the original ligand was located. The grid box was centered at the active binding sites with dimensions adjusted to the size of BO (center: 5.589, 54.534, 7.128; grid spacing: 0.375 Å; dimensions: 94, 110, 126 in x, y, and z directions, respectively). The docking was run with the ligand being flexible and the receptor being rigid. The number of GA runs was set to 200, and other parameters were set to the defaults.

Binding free energies (kcal/mol) were calculated based on the scoring function, and the pose with the lowest binding energy was selected for further analysis [52].

### 3.13. Molecular Dynamics Simulation

Molecular dynamics (MD) simulations were performed using the GROMACS 2024 software suite. The STAT3−BO complex was prepared using the LigPrep module within the Schrödinger molecular drug discovery suite (version 2018-1). The receptor protein and BO were parameterized using the AMBER ff14SB and GAFF2 force field parameters, respectively. BO was processed with the ante-chamber module, followed by the convert.py script to generate topology files, while STAT3 was prepared using the Leap module. The protein system to be simulated was then solvated in a solvent box containing the TIP3P water model, with boundaries set at least 10 Å away from the protein. An appropriate number of Cl^−^ ions was added to maintain electrical neutrality and complete the system setup. The constructed system underwent energy minimization using the mdrun module to remove unfavorable atomic contacts and prevent collapse during kinetic simulations. Following energy minimization, the system was pre-equilibrated under NVT and NPT conditions for 100 ps each, followed by simulations under NVT and NPT conditions at 300 K and 1 bar. Subsequently, a 50 ns molecular dynamics production run was conducted, with trajectories being saved every 2 fs. The gmx rms, gmx rmsf, gmx gyrate, gmx sasa, and gmx hbond modules in GROMACS were utilized to calculate the root-mean-square deviation (RMSD), root-mean-square fluctuation (RMSF), radius of gyration (Rg), solvent-accessible surface area (SASA), and hydrogen bond interactions of the protein based on simulation trajectories. Additionally, gmx_MMPBSA and gmx_MMPBSA_ana were employed to compute the binding free energy and residue decomposition. Various dynamic data were visualized using the Xmgrace software 5.1.25 [52].

### 3.14. Western Blot

Cells were seeded onto 60 mm plates at 5 × 10^6^ cells/well. After 24 h of treatment with 20 µM BO, the cells were washed with cold PBS, and the whole protein was extracted with RIPA lysis buffer containing protease inhibitor mixture. Equal amounts of proteins were separated using 10% sodium dodecyl sulfate–polyacrylamide gel electrophoresis and transferred onto a polyvinylidene fluoride (PVDF) membrane. The transferred protein was detected with STAT3 (1:1000 dilution, WL01836, Wanleibio, Shenyang, China) and GAPDH (1:5000 dilution, AF7021, Affinity, Changzhou, China) using a cECL Western Blot Kit. The bands were visualized and photographed using the e-Blot Touch Imager (eBlot, Shanghai, China).

### 3.15. Statistical Analysis

Data are presented as the mean ± standard deviation (SD). Statistical analysis was conducted using Student’s *t*-test or one-way analysis of variance (ANOVA) with GraphPad Prism 8 (La Jolla, CA, USA), and a *p*-value of less than 0.05 was regarded as statistically significant.

## 4. Discussion

BO is a cycloartane triterpene glycoside found in *A. vaginata*. The literature has revealed that there is limited research on BO to date. Based on our experimental results, compared with other cycloartane triterpene glycosides in *A. vaginata*, such as Soulieoside U, BO exhibits more significant therapeutic effects on breast cancer [53,54]. In in vitro pharmacological screening experiments, the IC_50_ values of BO for the two breast cancer cell lines MCF-7 and MDA-MB-231 were both less than 20 µM, which was significantly lower than the IC_50_ values observed in other cell lines (SW480, Huh7, 4T1, SHSY5Y, and HepG2). Previous studies indicated that concentrations above 20 µM are generally not considered to have pharmacological effects [54]. Based on these results, we inferred that BO exhibits the strongest cytotoxic activity against breast cancer cells. In the ADMET prediction of BO, BO demonstrated strong lipophilicity (low logS and high logP and logD values), and the MDCK permeability result for BO was “high passive permeability”, which supports BO’s ability to enter cells and exert its pharmacological effects. To further investigate the therapeutic effects of BO on breast cancer, we conducted more detailed phenotypic experiments using doses based on the IC_50_ values. In a colony formation assay, we used 1/2 × IC_50_, 1 × IC_50_, and 2 × IC_50_ for the low-, medium-, and high-dose groups, respectively. As the dose increased, BO showed significant inhibitory effects on the 14-day in vitro tumor formation of both MDA-MB-231 and MCF-7 cells. The higher the dose, the more pronounced the inhibitory effect. Since the threat of breast cancer to health primarily comes from distant metastasis, we chose the highly metastatic TNBC cell line MDA-MB-231 to conduct a transwell invasion assay to examine the impact of the drug on breast cancer metastasis. In the transwell assay, compared with the control group, the number of MDA-MB-231 cells that migrated to the lower chamber decreased with the introduction of the drug. Furthermore, as the dose of BO increased, the number of migrating MDA-MB-231 cells also decreased. Using the control group as a baseline, we calculated the relative migration rate of the treated groups, which showed a decreasing trend as the dose increased. This indicates that BO significantly inhibits the migratory and invasive abilities of MDA-MB-231 cells. In summary, we believe that BO exhibits therapeutic effects on breast cancer in multiple pathways.

STAT3 has indeed been identified as a key target in the occurrence, development, and drug resistance of breast cancer. The development of STAT3 inhibitors and degraders is a new direction for the treatment of breast cancer [55,56,57,58,59,60]. We predicted that BO treats breast cancer through STAT3 using network pharmacology, molecular docking, and molecular dynamics simulations, and we validated the predictions using Western blotting.

An aggregate of 179 potential targets of BO–breast cancer were identified through network pharmacology. A total of 172 of them formed a network, suggesting that the therapeutic effects of BO may be achieved through multi-target regulation, with possible synergistic effects on these targets [61]. Algorithms for the analysis of the MNC, MCC, and Degree were employed to analyze the 172 targets in the PPI network. After that, seven key targets were identified: HIF1A, STAT3, HSP90AA1, HSP90AB1, PIK3CA, MTOR, and NFKB1. Molecular docking studies were conducted on these seven targets to further identify the primary targets. To avoid inaccuracies caused by blind docking, we selected as many protein co-crystal structures with reported inhibitor ligands as possible from the PDB database. The molecular docking was performed by adopting a docking strategy that binds BO to the original ligand binding pocket [62]. In the docking of BO with seven target points, the binding energy of BO with STAT3 was lower than that of the other six targets at −6.78 kcal/mol. This indicates that among the seven key target points, the binding between BO and STAT3 is most stable, and the tendency to form a complex is most pronounced. Therefore, STAT3 was selected as the object for further research. To further explore the functional locations of these targets within cells, we examined their subcellular localization, focusing particularly on STAT3. In the GO enrichment of breast-cancer-related cellular components, the cytosol and nucleoplasm were identified among the top 10 significant cellular components. This means that treatment aimed at the components of the cytosol and nucleoplasm may have significant therapeutic effects. The cytosol and nucleoplasm closely overlap with STAT3’s subcellular localization. Thus, we have reason to believe that STAT3 plays a crucial role in BO’s therapeutic effect on breast cancer. We performed molecular dynamics simulations and calculated the RMSD, RMSF, Rg, SASA, LJ-SR energy, Coulomb-SR energy, GBSA, and PBSA to prove the reliability of the docking conformation. The experimental results show that the lowest-energy BO-STAT3 conformation obtained from molecular docking possesses good stability. Since the molecular docking used 6NUQ as the co-crystal structure of STAT3 with its degrader SI-109, BO’s docking occurred at the pocket site of SI-109, with good results. Therefore, we have reason to believe that BO is highly likely to be a STAT3-degrading agent as well [63]. The Western blot method was employed to verify the influence of BO on STAT3 levels. The results showed that compared with the control group, the expression levels of STAT3 in both cell types decreased after BO treatment. This indicates that BO can indeed reduce STAT3 levels, which corroborates the findings from molecular docking and dynamics simulations. Additionally, we utilized the R language to analyze data from the GEO database. The analysis revealed that there were no significant differences in STAT3 expression between TNBC and non-TNBC patients, suggesting that targeting STAT3 for breast cancer treatment could theoretically be effective in both TNBC and non-TNBC patients. This conclusion provides theoretical support for our in vitro experiments, where BO demonstrated therapeutic effects on both the non-TNBC cell line MCF-7 and the TNBC cell line MDA-MB-231.

Our study fills the pharmacological research gap concerning BO in the field of tumors for the first time by systematically interpreting the mechanism of BO’s treatment of breast cancer by regulating STAT3 through network pharmacology, molecular docking, molecular dynamics simulations, and experimental verification and, thus, providing a potential novel therapeutic agent for TNBC. However, this study still has some limitations. In the ADMET prediction of BO, while BO shows good hydrogen bond receptor and donor numbers and an appropriate number of rotatable bonds, its logP of 3.486 and MW of 662.4 exceed the range of the Lipinski rules. The complex multi-ring structure of BO results in poor water solubility and a large TPSA, which are unfavorable for oral administration. Additionally, as a saponin compound, safety is the primary limitation for the injectable administration of BO. Therefore, it is crucial to improve the structure of BO. We propose some hypotheses. For example, the sugar moiety at C-3 can be removed, and modifications can be made to the aglycone by introducing functional groups that enhance protein affinity. Additionally, the hydroxyl group at C-25 can also be modified. This study does not provide in vivo pharmacological and pharmacokinetic data for BO. This is primarily due to the difficulty in extracting BO. Although BO is also widely present in *Beesia calthifolia*, its extraction and purification processes remain complex [17]. Whether BO can be efficiently isolated is the most critical issue limiting research on it. Another question that needs to be explored in depth is whether BO, as a drug, also exerts inhibitory effects on other targets.

## 5. Conclusions

In this investigation, we demonstrated through in vitro screening, network pharmacology, molecular docking, and experimental validation that BO exerts its therapeutic effects on breast cancer via STAT3. BO can decrease the expression of STAT3 in human breast cancer cell lines. Compared with other reported components of *A. vaginata*, BO demonstrates significant potential for the development of highly effective medicine. Specifically, BO exhibits good activity against TNBC that is not possessed by other compounds from *A. vaginata.* Compared with other cycloartane triterpenoids, BO also has the advantages of being relatively easy to isolate and having better efficacy [64,65]. As the molecular docking shows, in the STAT3 protein, the formation of hydrogen bonds between Glu638, Tyr640, and the drug enhances the binding activity. The utilization of hydrogen bonding or hydrophobic interactions to fill the hydrophobic pocket region formed by Met 648, Tyr 640, Ile 653, and Pro639 can improve activity. This provides a clearer strategy for the development of STAT3 inhibitors. In addition, this study’s results reveal the mechanism of BO’s anti-breast-cancer activity for the first time. This provides a theoretical basis for the medicinal value of *A. vaginata* and fills the gap in this area of research. Based on the structure of BO, further research and optimization are beneficial for enriching the therapeutic options for TNBC and reducing the poor prognosis caused by the lack of treatment options. However, this study also has several limitations. The extraction and purification of BO are still highly inefficient and complex. This limits the application of BO. Moreover, the in vivo pharmacodynamic experiments of BO are still unexplored. More detailed physicochemical parameters and molecular optimization urgently need to be investigated.

## Figures and Tables

**Table 1:** The screening results of BO in different cell lines.

Cell Line	IC_50_ (μM)
SW480	>100
Huh7	>100
4T1	32.14
SHSY5Y	86.91
HepG2	21.17
MCF-7	13.83
MDA-MB-231	16.52

**Table 2:** The subcellular localization of hub targets.

Gene Name	Breast Cancer Cell Lines	Main Location
HSP90AA1	MCF-7, MDA-MB-231, SK-BR-3, et (*n* = 62)	Cytosol
HSP90AB1	MCF-7, MDA-MB-231, SK-BR-3, et (*n* = 62)	Cytosol
STAT3	MCF-7, MDA-MB-231, SK-BR-3, et (*n* = 62)	Nucleoplasm, Cytosol
PIK3CA	MCF-7, MDA-MB-231, SK-BR-3, et (*n* = 62)	Primary cilium, Cytosol, Microtubules, Mitochondria
HIF1A	MCF-7, MDA-MB-231, SK-BR-3, et (*n* = 62)	Nucleoplasm, Nuclear bodies
MTOR	MCF-7, MDA-MB-231, SK-BR-3, et (*n* = 62)	Golgi apparatus, Cytosol
NFKB1	MCF-7, MDA-MB-231, SK-BR-3, et (*n* = 62)	Nucleoplasm, Cytosol

**Table 3:** The docking results of hub targets.

Gene Name	PDB-ID	Energy kcal/mol
HIF1A	4H6J	−5.53
STAT3	6NUQ	−6.78
HSP90AA1	2YK9	>0
HSP90AB1	3NUQ	>0
PIK3CA	5DXT	>0
MTOR	4JSX	>0
NFKB1	1SVC	−6.55

**Table 4:** Free energies of BO with STAT3 calculated using the MM/GBSA method.

Differences (Complex–Receptor–Ligand):			
Energy Component	Average	Std. Dev.	Std. Err. of the Mean
VDWAALS	−30.78	2.56	0.25
EEL	−15.04	6.20	0.62
EGB	24.96	3.93	0.39
ESURF	−3.84	0.33	0.03
DELTA G gas	−45.83	7.14	0.71
DELTA G solv	21.12	3.70	0.37
DELTA TOTAL	−24.71	3.94	0.39

**Table 5:** Free energies of BO with STAT3 calculated using the MM/PBSA method.

Differences (Complex–Receptor–Ligand):			
Energy Component	Average	Std. Dev.	Std. Err. of the Mean
VDWAALS	−30.78	2.56	0.25
EEL	−15.04	6.20	0.62
EPB	28.03	3.88	0.39
ENPOLAR	−3.45	0.14	0.01
DELTA G gas	−45.83	7.14	0.71
DELTA G solv	24.58	3.80	0.38
DELTA TOTAL	−21.25	4.56	0.45

## Data Availability

The data presented in this study are available in this article (and Supplementary Materials). Additionally, other items that support the results of this study will be made available upon reasonable request.

## Supplementary Materials

The following supporting information can be downloaded at https://www.mdpi.com/article/10.3390/ijms26052283/s1.
