# Supplementary material for: Revealing the Role of Beesioside O from Actaea vaginata for the Treatment of Breast Cancer Using Network Pharmacology, Molecular Docking, and Molecular Dynamics Simulation
Source: Int J Mol Sci. 2025 Mar 4;26(5):2283. doi: 10.3390/ijms26052283 (PMC11899959; doi:10.3390/ijms26052283)
Supplement: Supplementary file 1 [file ijms-26-02283-s001.zip › S1 Cartesian coordinates of BO’s atoms.pdf]

Method to construct Cartesian coordinates of atoms

BO was drawn by ChemDraw 20.0 in the form of 2D structural data files (.cdx) and its 3D structural model was constructed using the ChemBio3D Ultra 14.0 program, followed by energy minimization with the MM2 forcefield, reaching a minimum RMS gradient of 0.01. Geometrical optimization and vibrational frequency calculations were conducted within the framework of Density Functional Theory (DFT), using the B3LYP/6–31 +G (d,p) basis set, supplemented with empirical dispersion corrections (EM=GD3BJ) by the Gaussian 16 Revision C.01 package. During optimization, default settings were applied for the convergence criteria of maximum force, RMS force, maximum displacement, and RMS displacement.

Result

1. BO minimum energy structure diagram

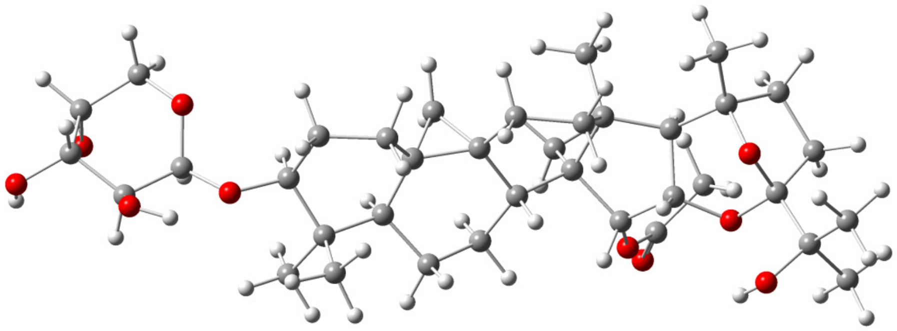

Figure S1. BO minimum energy structure diagram. It demonstrates the stereo structure of BO at energy minimization.

2. **Table S1.**Cartesian coordinates of atoms

Label	Serial Number	Atom Name	Alternate Location Indicator	X	Y	Z	Atom Name Repeat
HETATM	1	C	0	3.941	1.764	-0.044	C
HETATM	2	C	0	4.49	0.377	-0.409	C
HETATM	3	C	0	3.619	-0.398	-1.429	C
HETATM	4	C	0	2.257	-0.679	-0.699	C
HETATM	5	C	0	1.691	0.523	0.118	C
HETATM	6	C	0	2.401	1.858	-0.089	C
HETATM	7	C	0	1.175	-1.31	-1.592	C
HETATM	8	C	0	-0.042	-1.702	-0.746	C
HETATM	9	C	0	-0.758	-0.445	-0.229	C
HETATM	10	C	0	0.184	0.573	0.419	C
HETATM	11	C	0	-2.045	-0.728	0.628	C
HETATM	12	C	0	-2.617	0.651	1.278	C
HETATM	13	C	0	-2.049	1.851	0.479	C
HETATM	14	C	0	-0.506	1.944	0.518	C
HETATM	15	C	0	-4.163	0.439	1.135	C
HETATM	16	C	0	-4.274	-0.17	-0.259	C
HETATM	17	C	0	-3.179	-1.239	-0.368	C
HETATM	18	O	0	-5.599	-0.549	-0.612	O
HETATM	19	C	0	-6.525	0.588	-0.459	C
HETATM	20	O	0	-5.801	1.742	-0.103	O
HETATM	21	C	0	-5.315	1.512	1.256	C
HETATM	22	C	0	-6.566	0.871	1.942	C
HETATM	23	C	0	-7.409	0.311	0.771	C
HETATM	24	C	0	-4.986	2.875	1.848	C
HETATM	25	C	0	-7.232	0.776	-1.831	C
HETATM	26	C	0	-8.201	-0.392	-2.089	C
HETATM	27	C	0	-7.95	2.123	-1.921	C
HETATM	28	O	0	-6.235	0.799	-2.859	O
HETATM	29	C	0	-1.762	-1.787	1.71	C
HETATM	30	C	0	4.308	-1.747	-1.735	C
HETATM	31	C	0	-2.236	0.836	2.762	C
HETATM	32	C	0	1.174	0.191	1.505	C
HETATM	33	C	0	3.477	0.37	-2.761	C
HETATM	34	O	0	5.839	0.538	-0.914	O
HETATM	35	O	0	-3.481	-2.653	-0.285	O
HETATM	36	C	0	6.874	0.104	-0.093	C
HETATM	37	O	0	6.926	0.937	1.068	O
HETATM	38	C	0	7.847	0.458	2.048	C
HETATM	39	C	0	9.098	-0.211	1.448	C

HETATM	40	C	0	9.404	0.343	0.054	C
HETATM	41	C	0	8.189	0.206	-0.898	C
HETATM	42	O	0	8.197	1.334	-1.758	O
HETATM	43	O	0	10.584	-0.243	-0.474	O
HETATM	44	O	0	8.936	-1.634	1.276	O
HETATM	45	C	0	-4.213	-3.451	0.542	C
HETATM	46	C	0	-5.244	-2.873	1.482	C
HETATM	47	O	0	-4.024	-4.646	0.453	O
HETATM	48	H	0	4.373	2.49	-0.742	H
HETATM	49	H	0	4.323	2.03	0.946	H
HETATM	50	H	0	4.548	-0.24	0.497	H
HETATM	51	H	0	2.511	-1.454	0.041	H
HETATM	52	H	0	2.067	2.579	0.663	H
HETATM	53	H	0	2.089	2.277	-1.054	H
HETATM	54	H	0	1.571	-2.197	-2.098	H
HETATM	55	H	0	0.864	-0.608	-2.378	H
HETATM	56	H	0	0.293	-2.341	0.08	H
HETATM	57	H	0	-0.741	-2.309	-1.334	H
HETATM	58	H	0	-1.115	0.061	-1.14	H
HETATM	59	H	0	-2.382	1.809	-0.562	H
HETATM	60	H	0	-2.448	2.785	0.879	H
HETATM	61	H	0	-0.194	2.457	1.434	H
HETATM	62	H	0	-0.192	2.59	-0.312	H
HETATM	63	H	0	-4.395	-0.337	1.87	H
HETATM	64	H	0	-3.959	0.605	-0.967	H
HETATM	65	H	0	-2.789	-1.214	-1.387	H
HETATM	66	H	0	-7.124	1.64	2.483	H
HETATM	67	H	0	-6.287	0.103	2.667	H
HETATM	68	H	0	-7.644	-0.752	0.862	H
HETATM	69	H	0	-8.353	0.855	0.68	H
HETATM	70	H	0	-4.243	3.41	1.257	H
HETATM	71	H	0	-4.615	2.774	2.872	H
HETATM	72	H	0	-5.894	3.485	1.873	H
HETATM	73	H	0	-8.604	-0.294	-3.101	H
HETATM	74	H	0	-9.038	-0.395	-1.384	H
HETATM	75	H	0	-7.684	-1.354	-2.015	H
HETATM	76	H	0	-8.417	2.21	-2.906	H
HETATM	77	H	0	-7.238	2.941	-1.796	H
HETATM	78	H	0	-8.732	2.214	-1.161	H
HETATM	79	H	0	-5.733	-0.025	-2.777	H
HETATM	80	H	0	-0.917	-1.488	2.331	H
HETATM	81	H	0	-1.532	-2.756	1.264	H
HETATM	82	H	0	-2.615	-1.933	2.376	H

HETATM	83	H	0	4.471	-2.331	-0.821	H
HETATM	84	H	0	5.28	-1.581	-2.209	H
HETATM	85	H	0	3.709	-2.356	-2.418	H
HETATM	86	H	0	-2.562	1.822	3.111	H
HETATM	87	H	0	-1.157	0.776	2.921	H
HETATM	88	H	0	-2.708	0.09	3.407	H
HETATM	89	H	0	1.336	0.91	2.306	H
HETATM	90	H	0	1.244	-0.846	1.825	H
HETATM	91	H	0	2.92	1.305	-2.665	H
HETATM	92	H	0	2.964	-0.244	-3.507	H
HETATM	93	H	0	4.468	0.609	-3.161	H
HETATM	94	H	0	6.712	-0.937	0.227	H
HETATM	95	H	0	7.348	-0.255	2.723	H
HETATM	96	H	0	8.138	1.338	2.631	H
HETATM	97	H	0	9.961	-0.023	2.1	H
HETATM	98	H	0	9.607	1.414	0.14	H
HETATM	99	H	0	8.299	-0.719	-1.485	H
HETATM	100	H	0	7.291	1.453	-2.081	H
HETATM	101	H	0	10.49	-1.203	-0.383	H
HETATM	102	H	0	9.036	-2.079	2.127	H
HETATM	103	H	0	-4.76	-2.414	2.349	H
HETATM	104	H	0	-5.866	-3.697	1.835	H
HETATM	105	H	0	-5.849	-2.121	0.979	H
